# Spatiotemporal distribution of SARS-CoV-2 vaccines and vaccine-related proteins in mice and humans

**DOI:** 10.1038/s41598-026-47568-6

**Published:** 2026-05-19

**Authors:** Fabian Heinrich, Jöran Lücke, Siwen Zhang, Morsal Sabihi, Christian Bernreuther, Katja Giersch, Lena Allweiss, Kristin Hartmann, Edda Thies, Philine Lange, Jakob Matschke, Anja A. Kühl, Ronja Mothes, Helena Radbruch, Ann Sophie Schröder, Axel Heinemann, Maura Dandri, Martin Aepfelbacher, Samuel Huber, Anastasios Giannou, Markus Glatzel, Benjamin Ondruschka, Marc Lütgehetmann, Susanne Krasemann

**Affiliations:** 1 Institute of Legal Medicine, University Medical Center Hamburg-Eppendorf, Butenfeld 34, 22529 Hamburg, Germany; 2 Department of Medical Statistics, London School of Hygiene and Tropical Medicine, London, UK; 3 Centre for Data and Statistical Science for Health, London School of Hygiene and Tropical Medicine, London, UK; 4 Section of Molecular Immunology and Gastroenterology, I. Department of Medicine, University Medical Center Hamburg-Eppendorf, 20246 Hamburg, Germany; 5 Hamburg Center for Translational Immunology (HCTI), University Medical Center Hamburg-Eppendorf, 20246 Hamburg, Germany; 6 Department of General, Visceral and Thoracic Surgery, University Medical Center Hamburg-Eppendorf, 20246 Hamburg, Germany; 7 Institute of Pathology, University Medical Center Hamburg-Eppendorf, Martinistraße 52, 20251 Hamburg, Germany; 8 Institute of Medical Microbiology, Virology and Hygiene, University Medical Center Hamburg-Eppendorf, Martinistraße 52, 20251 Hamburg, Germany; 9 Department of Internal Medicine, University Medical Center Hamburg-Eppendorf, Martinistraße 52, 20251 Hamburg, Germany; 10 German Center for Infection Research (DZIF), Hamburg-Lübeck-Borstel-Riems site, Hamburg, Germany; 11 Institute of Neuropathology, University Medical Center Hamburg-Eppendorf, Martinistraße 52, 20251 Hamburg, Germany; 12 Charité – Universitätsmedizin Berlin, Berlin, Germany; 13 Department of Neuropathology, Charité-Universitätsmedizin, Berlin, Germany

**Keywords:** Vaccination, SARS-CoV-2, COVID-19, mRNA vaccine, Spatiotemporal distribution, Biological techniques, Immunology

## Abstract

Since the emergence of the COVID-19 pandemic, the successful distribution and application of mRNA vaccines have helped to contain the spread of SARS-CoV-2 and saved countless lives worldwide. mRNA vaccines are not only being developed and tested for infectious diseases, but also for numerous new applications, such as cancer therapy. Although the general mechanisms of mRNA vaccinations have been thoroughly studied, data regarding the local anatomical distribution after vaccination have been scarce. Here, we investigated the spatiotemporal distribution of BNT162b2 and mRNA-1273 vaccines in mice and deceased humans. We found that vaccine-related mRNA and spike protein could be detected at the vaccination site of patients and mice, with muscle-associated fibroblasts being a major source of spike protein expression. In contrast, we did not detect expression of vaccine-related spike protein in immune cells at the injection site. While mRNA-vaccine-related mRNA or spike protein could not be detected in extramuscular organs in humans in our study, it was detected in several organs in mice, even up to 7 days after initial vaccination. Together, these data enhance our understanding of mRNA vaccine kinetics and distribution patterns, providing valuable insights to guide scientists in refining future mRNA vaccine-based therapies whenever appropriate.

**Supplementary Information:**

The online version contains supplementary material available at 10.1038/s41598-026-47568-6.

## Introduction

In 2019, the worldwide emergence of SARS-CoV-2 led to a long-lasting pandemic^1^. Over the following years, it led to hundreds of millions of infections and millions of deaths^2^. However, global initiatives have successfully developed safe and effective mRNA vaccines targeting SARS-CoV-2, thereby limiting the extent of the COVID-19 pandemic by inducing immune protection^3^. With mRNA vaccines first introduced in 2005^4^, the COVID-19 pandemic presented the first opportunity to conduct phase II and III multicentre trials on mRNA vaccine efficacy^5,6^. Indeed, studies showed a high efficacy of > 94% against symptomatic COVID-19 in the first month after vaccination^7^. Safety profiles from these studies indicate that mRNA vaccines were generally well-tolerated, with adverse events typically mild to moderate and transient^5^. Injection site pain, as well as swelling or redness, was observed in up to 89% of recipients. In contrast, treatment-related side effects were reported in 8 to 21% of recipients, most commonly fatigue and headache^5,6^. Until 2021, the most used mRNA vaccines against SARS-CoV-2 were developed by BioNTech (BNT162b2^5^) and Moderna (mRNA-1273^6^). Both mRNAs aim to produce spike protein, a membrane protein of SARS-CoV-2^8^, and are packaged within lipid nanoparticles (LNPs) to enable delivery into target cells. Now that the COVID-19 pandemic has significantly slowed its course, new applications of mRNA vaccines are being tested. The potential of nucleic acid-based vaccines is currently explored to increase the efficacy of conventional vaccines against seasonal influenza^9^. Especially the unique feature of nucleic acid-based vaccines to express membrane-bound protein versions makes them attractive for their application to develop alternative vaccination strategies for other viral diseases such as the human immunodeficiency virus HIV-1^10–12^. Moreover, multiple studies are currently investigating the treatment of cancer by mRNA vaccination^13^. Given the success story of mRNA vaccines during the COVID-19 pandemic^14^, they are likely to have a significant impact in the future.

Although the immunological response to vaccination with conventional vaccines has been studied for decades and is generally well understood^15^, mRNA vaccination processes are still less well understood. They were optimised for uptake by antigen-presenting cells in vitro using cell-culture experiments. Initial studies hypothesised that mRNA vaccines are rapidly transported from the injection site to the draining lymph nodes and are predominantly taken up by antigen-presenting cells that subsequently express the protein of the encoded antigen^16,17^. A recent in vivo imaging study in nonhuman primates has confirmed this model, demonstrating that, following vaccination, LNP/ mRNA are transported from the injection site to nearby lymph nodes within 4 h and in lower quantities even to the liver^18^. Interestingly, a recent study showed the enrichment of vaccine derived mRNA in fibroblasts within the injected muscle tissue in experimental mice using single cell analysis^19^. Moreover, this study showed that specifically the vaccine mRNA, and not just the LNPs, could induce an IFN-β-response in those fibroblasts, followed by recruitment of immune cells to the injection site^19^.

However, specific information regarding the persistence of mRNA vaccines and their derived proteins locally and in different organs remains scarce, especially in humans. Nonetheless, a comprehensive understanding of the biodistribution and expression profiles of mRNA vaccines and their derived proteins is essential for understanding the basis of their superior immunogenicity, to characterise differences in immune activation compared to conventional vaccine platforms, and to further improve their future applications.

In this study, we investigated the spatiotemporal distribution of two SARS-CoV-2 mRNA vaccines, namely BNT162b2 and mRNA-1273, and their respective vaccine-derived spike protein in mice and in human tissues. In mice, vaccine mRNA could be found in numerous organs, including the injection site muscle tissue, but was rapidly cleared within days. In contrast, in autopsy tissue of human individuals, vaccine mRNA was almost exclusively detected at the injection site. Immunohistochemical detection of vaccine-derived spike protein showed that the majority of spike protein in the muscle of both mice and humans could be found in fibroblasts and to a lesser degree in muscle stem cells, i.e. satellite cells. Our findings guide future vaccine development and administration strategies.

## Materials and methods

### Population

#### Animals

Six- to eight-week-old male and female C57BL/6 mice weighing 18 to 25 g were used. All mice were bred and housed under pathogen-free conditions in the University Medical Center Hamburg Eppendorf animal facility. All animal experiments were carried out in accordance with relevant guidelines and regulations and were reviewed by the Institutional Review Board “Behörde für Justiz und Verbraucherschutz, Lebensmittelsicherheit und Veterinärwesen” (Hamburg, Germany, N014/2022). All methods involving animal experiments are reported in accordance with ARRIVE guidelines.

#### Humans

The Institute of Legal Medicine registered all known death cases in a temporal relationship with COVID-19 vaccination as part of a death case evaluation during the pandemic era to support a comprehensive overview for local health authorities. Autopsies were requested based on the Infection Protection Act and by physicians and relatives. All patients admitted with death in a temporal relationship with a COVID-19 vaccine, defined as a maximum of 14 days after vaccination, were enrolled in this study. All patients aged > 18 years were included between January and December 2021 in accordance with relevant guidelines and regulations. The Hamburg Chamber of Physicians Ethics Committee approved this study (2020-10353-BO-ff).

Patients with active and/ or past COVID-19 diagnosis were excluded from the study. An active and/ or past COVID-19 diagnosis was ruled out based on anamnesis, negative SARS-CoV-2 qPCR and serological testing. Nasopharyngeal swabs were taken as part of the clinical routine at admission to the Institute of Legal Medicine. Reverse transcription-quantitative polymerase chain reaction for SARS-CoV-2 RNA was performed as described previously at the Institute of Medical Microbiology, Virology and Hygiene (University Medical Center Hamburg-Eppendorf, Hamburg, Germany)^20^. Serum blood samples were obtained while performing a full autopsy. Qualitative antibody levels directed against SARS-CoV-2 nucleocapsid protein were examined using Elecsys Anti-SARS-CoV-2 (Roche Diagnostics International Ltd, Rotkreuz, Switzerland). Quantitative antibody levels directed against SARS-CoV-2 spike protein were detected using Elecsys Anti-SARS-CoV-2-S (Roche Diagnostics International Ltd). All assays were used as recommended by the manufacturer on the appropriate automated device (Cobas e411, Roche Diagnostics International Ltd). Cut-off values were set according to the manufacturer’s recommendations: >1.0 COI (Elecsys Anti-SARS-CoV-2) and > 0.8 U/ml (Elecsys Anti-SARS-CoV-2-S). To rule out post mortem degradation of SARS-CoV-2 antibodies, Spearman correlation coefficients were calculated, in which no evidence of a non-linear monotonic relationship between the time from death until admission and SARS-CoV-2 antibody levels was observed (Elecsys Anti-SARS-CoV-2-S: *r* = 0.01, *p* = 0.79). Informed consent was obtained from the relatives and legal representatives of the deceased.

### Exposure

#### Animals

Mice received a single intramuscular injection of 50 µL vaccine solution into the medial gastrocnemius muscle on the right side with a 30G cannula. For this injection, the mice were anaesthetised using isoflurane in a concentration of 2%. The injection site and the corresponding area on the contralateral side were shaved and disinfected with 96% ethanol. The 50 µL injection solution contained either 1 µg *BNT162b2* or 5 µg of *mRNA-1273* from commercially available preparations and was diluted accordingly with PBS. Dosages were determined according to the corresponding animal approval studies^21,22^.

#### Humans

Patients vaccinated with COVID-19 mRNA vaccines. BNT162b2 and mRNA-1273 were administered according to national guidelines as part of routine clinical care. Dosages were determined according to the corresponding national recommendations.

### Outcome

#### Animals

Vaccine mRNA and spike protein distribution were investigated in multiple organs. Animals were sacrificed 1, 2, 3, 4, 5, and 7 days after the vaccination. For this, mice were anaesthetised using isoflurane in a concentration of 2% followed by cervical dislocation. Muscle tissue where the injection was administered, along with its draining lymph node, as well as the contralateral uninjected muscle and lymph node, blood, heart, kidney, liver, spleen, and the right and left lungs, were collected. Organ samples were immediately stored in TRIzol at -80 °C or fixed in 4% paraformaldehyde at room temperature. The presence and quantity of vaccine-derived mRNA were assessed using quantitative reverse-transcription polymerase chain reaction (RT-qPCR), while the detection of spike protein was determined via immunohistochemistry (IHC), as described below. Vaccine-derived mRNA and spike protein distribution were stratified by the time after vaccination. The contralateral gastrocnemius muscle served as an internal control tissue.

#### Humans

Vaccine mRNA and spike protein distribution were investigated in multiple organs. Conventional autopsies were performed at the Institute of Legal Medicine of the University Medical Center Hamburg-Eppendorf, Hamburg, Germany. All post mortem examinations were performed following guidelines from the German Society of Legal Medicine. During the autopsy, tissue samples of 30 mm^3^ were sampled and stored, including the deltoid muscle, axillary lymph nodes, inguinal lymph nodes, spleen, liver, kidney, and lung. Blood samples were taken from the superficial femoral vein. Tissue samples were immediately stored at -80 °C or in 4% paraformaldehyde at room temperature. Again, mRNA was determined using quantitative reverse-transcription polymerase chain reaction (RT-qPCR), as described below, and spike protein was detected using immunohistochemistry (IHC) as described below. Vaccine mRNA and spike protein distribution were stratified by the time after vaccination. The date and site of vaccination were obtained using anamnesis or clinical records. Vaccine mRNA and spike protein distribution were stratified by the time after vaccination. The contralateral deltoid muscle served as an internal control tissue.

### Quantitative RT-PCR for BNT162b2 and mRNA-1273 mRNA in human tissues

All human tissue samples were processed using 2 mL microtubes prefilled with ceramic beads (Precellys lysing Kit, Bertin Technologies SAS, Montigny-le-Bretonneux, France) and 1 mL phosphate-buffered saline for tissue homogenisation on the Precellys 24 Tissue Homogeniser (Bertin Technologies SAS, Montigny-le-Bretonneux, France). Blood samples were diluted as needed using 0.9% sodium chloride (B. Braun SE, Hessen, Germany). For RNA/DNA extraction, 200 µL of the homogenised tissue lysate/ whole blood was transferred to the MagNA Pure96 (Roche Molecular Systems, CA, USA). Automated nucleic acid extraction was performed according to the manufacturer’s recommendations with whole process control (Roche Control Kit). To monitor the nucleic acid purification and detection processes and to prevent false-negative results, 20 µL of internal control RNA (IC-RNA; Roche Control Kit) were automatically added to each individual sample during the extraction procedure. The final elution volume amounted to 100 µL. BNT162b2-RNA-specific primers and probes were designed at the nucleotide positions in the S gene of SARS-CoV-2, in total alignment with the synthetic construct SARS_CoV_2_ectoCSPP gene (GenBank number: MT380725.1)^23^. One-step reverse transcription PCR was run on the LightCycler 480 system (Roche Diagnostics International Ltd) by using the S-gene forward (5´-TCT ACC CAG GAC CTG TTC CTG C, 400 nM) and reverse primer (5´-GGG CAG CAC GGG GTT GTC GAA T, 400 nM) and probe (5´ FAM -TGG TGC CGG ACA CGT GGA TGG CGT-BHQ1, 100 nM), and a one-step RNA control kit (Roche Diagnostics International Ltd) as master mix. Briefly, the RT-qPCR master mix consisted of 0.1 µL RT Enzyme Solution, 4.0 µL RT-qPCR Reaction Mix, 1.0 µL RNA Process Control Detection Assay, 1.0 µL forward primer, 1.0 µL reverse primer, 0.5 µL probe, 7.4 µL PCR-grade water, and 5.0 µL RNA template. Thermal cycling conditions were as follows: reverse transcription at 50 °C for 10 min (slope 4.4); pre-incubation at 95 °C for 30 s (slope 4.4); amplification for 45 cycles consisting of denaturation at 95 °C for 5 s (slope 4.4) and annealing/extension at 60 °C for 30 s with single fluorescence acquisition (slope 2.2); followed by cooling at 40 °C for 30 s (slope 2.2). Fluorescence signals were detected in each amplification cycle in the FAM and Cy5 channels using the 3-colour hydrolysis probe filter setting, as recommended by Roche. Ct values were determined using the second derivative maximum method implemented in the LightCycler software (version 5.1). Detection of S-RNA was defined as a positive result by the presence of a sigmoidal amplification curve with a Ct value < 40 in the target channel (FAM). Negative samples were defined by the absence of a sigmoidal amplification curve in the target channel (FAM) in combination with a valid amplification curve in the internal control (IC) channel (Cy5) with Ct values between 30 and 35. Samples lacking an IC signal were classified as invalid. If sufficient material was available, invalid samples were reanalysed, starting the process anew from tissue disruption. Positive and negative controls were included in each RT-qPCR run. Amplification curves were independently reviewed by two trained operators. The samples were analysed once if a valid result (positive or negative) was obtained. For quantification, RNA was extracted from the commercially available BNT162b2 vaccine (BioNTech SE, Mainz, Germany), and absolute copy numbers were determined by droplet digital PCR (ddPCR) using the one-step RT-ddPCR Advanced Kit for probes (Bio-Rad, California, USA) on the Bio-Rad QX100 droplet digital PCR system. A standard curve was prepared in 1:10 dilution steps (*n* = 5) (Figure S1). BNT162b2 RNA copy numbers are given. Due to substantial inter-individual variability in postmortem tissue properties, tissue-specific limits of detection could not be experimentally determined. For tissue samples, an initial input corresponding to 10 mg (approximately 10⁶–10⁷ human cell equivalents) was targeted in order to avoid saturation of the extraction chemistry. As internal control RNA signals were considered acceptable up to 5 Ct below the expected value (corresponding to an under-quantification of approximately 1.5 log), we estimate that, under the applied conditions, the analytical sensitivity of the assay is on the order of 5 × 10⁴ copies per 10 mg human cell equivalents or per 1 mL of postmortem blood. This value represents an approximation rather than a formally validated tissue-specific LOD.

### Quantitative RT-PCR for *BNT162b2* and *mRNA-1273* mRNA in mice

Tissues were homogenised using 2 mL microtubes prefilled with ceramic beads (Precellys lysing Kit, Bertin Technologies SAS, Montigny-le-Bretonneux, France) and 1 mL TRIzol Reagent (Invitrogen) for tissue homogenisation on the Precellys 24 Tissue Homogeniser. Thereafter, RNA was solubilised and purified through phase separation with chloroform, followed by isopropanol precipitation. The RNA pellet was washed with 75% ethanol, air-dried, and resuspended in RNase-free water. Next, the High-Capacity cDNA Synthesis Kit (Applied Biosystems) was used for cDNA synthesis. The following primers were used to detect vaccine-derived mRNA (BNT162b2: forward: 5′-TACCAAGCTGAACGACCTGT-3′, reverse: 5′-TTGCTGTTCCAGGCAATCAC-3′; mRNA-1273: forward: 5′-CGATCTCTTGTAGATCTGTTCTC-3′, reverse: 5′-GGTGAACCAAGACGCAGTAT-3′)^24,25^. Real-time PCR was performed using the Power SYBR^®^ Green PCR Master Mix (Thermo Fisher Scientific) on the StepOne Plus system (Applied Biosystems). Finally, relative expression was normalised to *Gapdh* and determined with the 2^−ΔΔCt^ method. In mice, an indicated RNA sequence was considered detectable when a value above or equal to 0.1 was measured, and undetectable when the value was below 0.1. In this case, the relative RNA expression was set to a detection limit of 0.0001. The threshold value was validated using positive and negative controls.

### Expression of the BNT162b2 spike protein in cultured cells

An aliquot of 50 µL of commercially available BNT162b2 was added to human HEK cells (confluency of 80%) to validate antibodies for the detection of vaccine-related spike protein (Figure S2B). Cells were fixed after 2 days in culture. Subsequently, cells were permeabilised with Triton-X100 at 0.2% in PBS for 5 min, blocked using MAXblock (ActiveMotif, CA, USA), and the BNT162b2 vaccine-related spike protein was stained overnight with different anti-spike antibodies. Specific binding was visualised by indirect detection using a fluorescently labelled secondary anti-mouse antibody, and two antibodies with the best signal-to-noise ratio were selected for all further experiments (anti-SARS-CoV-2 Spike (#GTX632604 and #GTX135356; GeneTex, Irvine, USA). Nuclei were counterstained with DAPI. Representative images were taken on a Leica SP5 Confocal microscope with a 40x objective. Alternatively, primary human muscle satellite cells (#P10976, InnoProt, Bizkaia, Spain) were incubated with 50 µL of commercially available BNT162b2, and spike protein was detected as described above after 48 h.

### In-situ hybridisation for BNT162b2 mRNA (RNAscope^®^) in humans

Vaccine mRNA was detected in paraffin sections with the RNAscope^®^ 2.5 HD Duplex Detection Kit (Advanced Cell Diagnostics, Newark, California, USA) following the manufacturer’s instructions. BNT162b2 mRNA (synthetic construct SARS_CoV_2_ectoCSPP gene; GenBank number: MT380725.1) was detected with a specific probe (RNAscope^®^ Probe - syn-SARSCoV-2-ectoCSPP-C1; #1117951-C1). A probe for the housekeeping gene GAPDH was used to assess RNA integrity (#442201-C1), and the RNAscope^®^ 3-plex Negative Control Probe (DapB; #320871) was used as a negative control (Figure S2B). RNAscope was performed on deltoid muscle tissue from two individuals with the highest vaccine mRNA levels, as determined by PCR, to assess the distribution of vaccine mRNA. Transcript levels of the housekeeping gene GAPDH were comparable between the two samples, although lower than those of the vaccine mRNA. This is not surprising given the high levels and stability-enhancing modifications of the injected mRNA.

### Immunohistochemistry and immunofluorescence for vaccine-related spike protein and immunological markers in mice and humans

Immunohistochemical stainings were performed using a Ventana BenchMark XT autostainer (Roche Diagnostics Deutschland GmbH, Mannheim, Germany) as previously described^26^. The specificity of anti-SARS-CoV-2 spike antibodies had been thoroughly tested previously^26^. In addition, we performed double staining of the most specific anti-SARS-CoV-2 spike protein antibody (#GTX632604) with an anti-SARS-CoV-2 nucleocapsid protein directed antibody (#40143-T62; SinoBiologica) in tissue of a patient with fatal COVID-19 to confirm specificity and proper sub-cellular localisation of spike protein signal (Figure S2A). Moreover, in every round, negative controls were included using the contralateral muscle and/or applying secondary-antibody-only and isotype control antibodies on tissues (Mouse IgG1 Isotype Control, #14-4714-82; eBiosciences, Inc., SanDiego, USA) (Figure S2D + E). Antibodies used in our study were directed against SARS-CoV-2 Spike (#GTX632604 and #GTX135356; GeneTex, Irvine, USA) or HLA-DR/DP/DQ (clone CR3/43, #M0775; Dako/ Agilent, Santa Clara, USA), CD68 (#M0876; Dako/ Agilent), CD8 (clone SP16; #ab101500; Abcam, Cambridge, UK), and CD3 (clone SP7, #ZYT-RBK024-05; Biozol, Germany). Representative images were taken with a Zeiss Axioscope 5 microscope and Axiocam 208 color camera. Immunofluorescence staining was performed as previously described^27^. Antibodies used for protein-specific detection were SARS-CoV-2 Spike and HLA-DR/DP/DQ or IBA1 (#HS-234308; Synaptic Systems, Göttingen, Germany) or CD14 (#ab133335; Abcam). Detection was performed using fluorescently labelled secondary anti-rabbit, -guinea pig, and -mouse antibodies, respectively. The tyramide signal amplification (TSA) method was used to perform double staining of spike protein and HLA-DR/DP/DQ or CD56 according to the manufacturer’s recommendations (TSA Plus Fluorescein; #NEL741001KT; Akoya Biosciences, Marlborough, USA). Representative images were taken on a Leica SP5 Confocal microscope (provided by UMIF; UKE).

Abundance of spike^+^-cells was assessed in five different fields of view (each 273000 µm^2^) per mouse muscle in a semi-quantitative manner. Values for scoring were defined as follows: 0 (no spike^+^-cells detectable), 1 (single positive cells with a total of 1–5 spike^+^-cells detectable only within one field of view), 2 (> 5 spike^+^-cells detectable sometimes in small groups in several fields of view), 3 (numerous spike^+^-cells detectable with several cluster of positive cells within different fields of view). Scoring was performed blinded to the experimental groups by an experienced pathologist.

### Statistical analysis

Continuous variables were summarised using mean (SD) and median (IQR), as appropriate. Continuous variables were inspected for approximate normality using histograms. Categorical variables were summarised as numbers (%). Scatter plots were used to illustrate the data. Means and 95% confidence intervals are shown. GraphPad Prism software version 9.1.1 (GraphPad Software, CA, USA) was used for data illustration. Data curation and analysis were conducted using STATA/MP, Version 17.0 (StataCorp, Texas, USA).

## Results

### Temporal dynamics of BNT162b2 and mRNA-1273 mRNA and spike protein at the vaccination site and different organs in mice

**Fig. 1:**
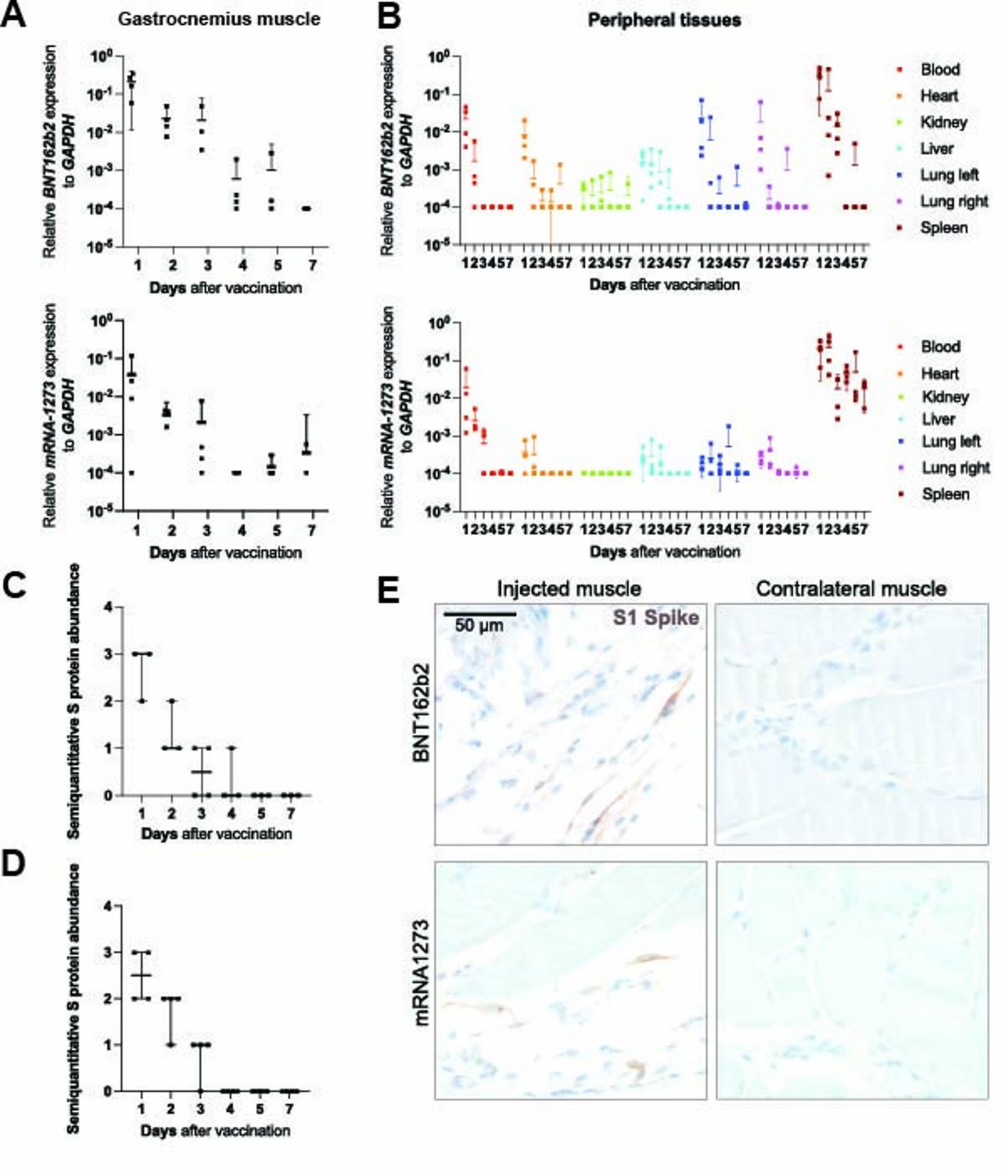
Temporal dynamics of *BNT162b2*-RNA, *mRNA-1273*-RNA, and spike protein in muscle following vaccination in mice. **(A)** The relative *BNT162b2* (upper panel) and *mRNA-1273* (lower panel) associated RNA expression in comparison to *Gapdh* at the vaccine injection site (medial gastrocnemius muscle) of mice (*n* = 2–4 per group) after indicated time points post vaccination was determined by qPCR. Data are pooled from two independent experiments. Horizontal lines represent means and 95% confidence intervals; each symbol indicates one tissue sample from one mouse. (**B)** The relative *BNT162b2* (upper panel) and *mRNA-1273* (lower panel) associated RNA expression in comparison to *Gapdh* in major tissues and organs of mice (*n* = 2–4 per group) after indicated time points post intra-muscular vaccination was determined by qPCR. Data are pooled from two independent experiments. Horizontal lines represent means and 95% confidence intervals; each symbol indicates one tissue sample from one mouse. **(C**,** D**) Semi-quantitative abundance of (**C**) *BNT162b2*- or (**D**) *mRNA-1273*-related spike (S) protein was determined by IHC at the vaccine injection site (right *medial gastrocnemius muscle*) of mice (*n* = 2–4 per group) after indicated time points. Data are pooled from two independent experiments; each symbol indicates one tissue sample from one mouse. (**E**) *BNT162b2*- (upper panel) and *mRNA-1273*- (lower panel) related spike protein expression at the vaccine injection site (right *medial gastrocnemius muscle*) of mice was examined 1 day following vaccination by IHC on formalin-fixed paraffin-embedded sections. Spike protein was detected in fibroblasts and satellite cells, respectively. The contralateral non-injected muscle served as a negative control and stayed immuno-negative.

The development of mRNA-based vaccines has been a game-changer in the fight against COVID-19. However, as with any novel technology, knowledge gaps remain. To obtain a temporal impression of vaccine kinetics at the vaccination site and in various organs, we intramuscularly injected mice with either BNT162b2 or mRNA-1273 into the right medial gastrocnemius muscle. High *BNT162b2* mRNA levels were detectable within the injected muscle tissue by qPCR one day after vaccination. Its abundance decreased over the following days, and by seven days after the initial vaccination, *BNT162b2* vaccine mRNA was no longer detectable (Fig. 1A). After vaccine administration, *BNT162b2* mRNA was detected widely in the peripheral organs, with pronounced abundance observed in the spleen. However, its levels decreased below the detection limit by days 3 to 4 (Fig. 1B). Following *mRNA-1273* vaccination, mRNA kinetics in the right medial gastrocnemius muscle were highest one day after injection, subsequently decreasing day by day (Fig. 1A). Meanwhile, mRNA abundance in peripheral organs was primarily confined to the spleen, remaining consistently high until day 7 (Fig. 1B). To investigate if the vaccine-derived mRNA is translated into protein at the injection site, we assessed the abundance of spike protein expression in muscle tissue by immunohistochemistry (IHC). In accordance with *BNT162b2* mRNA abundance, spike protein expression in muscle tissue at the vaccination site was detected and peaked as early as day one (Fig. 1C). Spike protein-positive cell detection decreased during the following time points, and could only be detected in one sample on day 4, but not at later time points post injection of mRNA vaccine (Fig. 1C). A similar spike protein kinetic was detected in the muscle tissue after vaccination with *mRNA-1273* (Fig. 1D). When investigating spike protein expression after *BNT162b2* or *mRNA-1273* vaccination via IHC, we found that fibroblasts and satellite cells were a significant source of the spike protein (Fig. 1E, Figure S3A). In summary, similar kinetics of vaccine-related mRNA and spike protein abundance in the right medial gastrocnemius muscle were observed in mice following vaccination with both mRNA-based vaccines investigated, BNT162b2 and mRNA-1273. Besides fibroblasts, satellite cells presented a source for spike protein expression in the injected muscle in mice.

### BNT162b2 vaccination leads to spike protein expression in muscle tissue but no long-lasting extra-muscular detection of *BNT162b2* mRNA or spike protein in humans

**Fig. 2:**
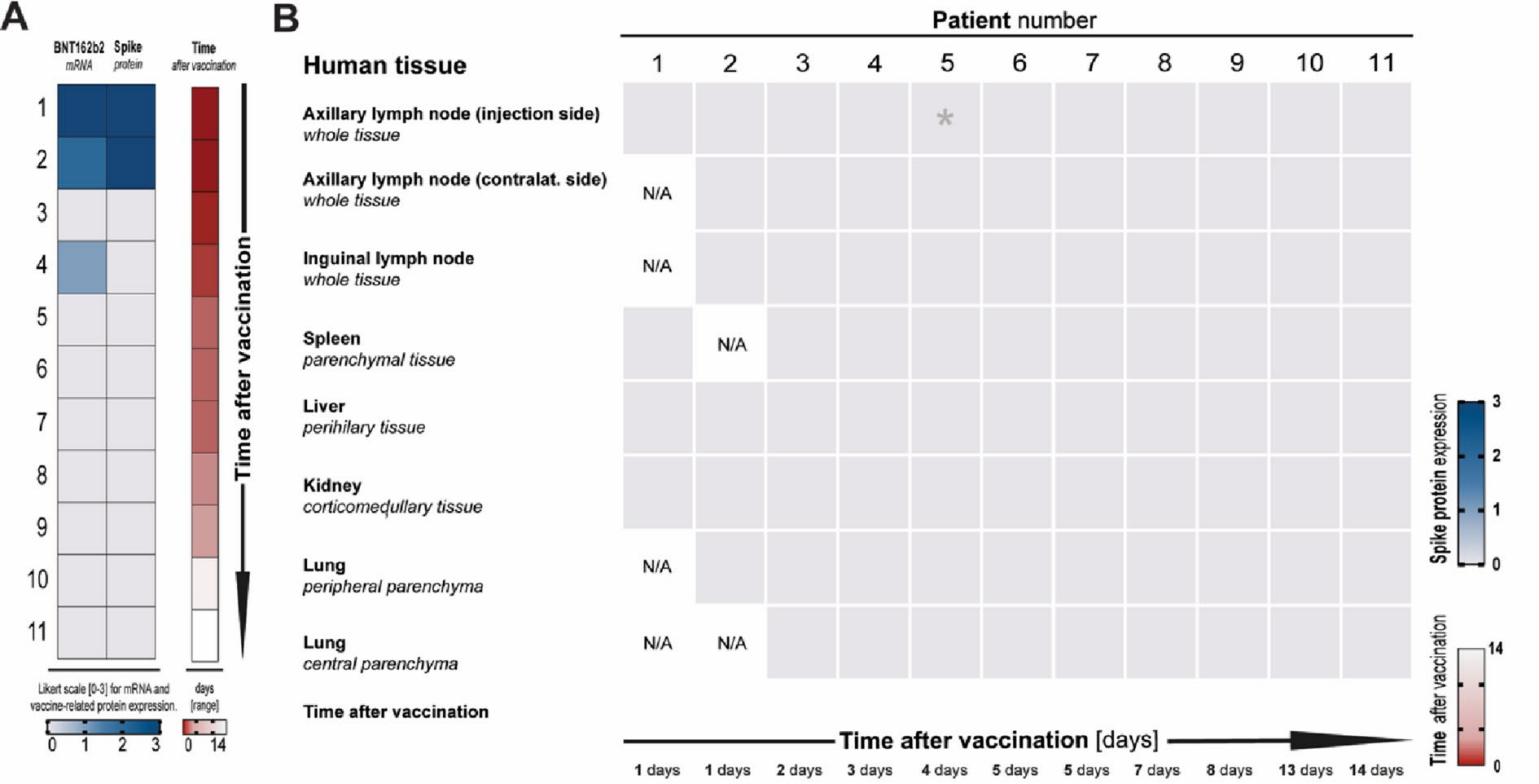
Organ distribution of vaccine-specific BNT162b2 mRNA and spike protein is restricted to human deltoid muscle after vaccination. (**A)** The semiquantitative abundance of BNT162b2-related mRNA and spike protein, determined by RT-qPCR and in-situ hybridisation or IHC, respectively, at the vaccine injection site (deltoid muscle) in eleven deceased patients in temporal relation to vaccination with *BNT162b2*. Blue squares represent a positive signal, and grey squares depict muscle tissues without detection of mRNA or spike protein. Cases were sorted by time after vaccination (in days, see Supplementary Table 1 for patient characteristics). (**B)** The indicated organs from all 11 patients vaccinated with *BNT162b2* were examined by RT-qPCR from cryopreserved samples; one qPCR-positive tissue was indicated by an asterisk (*). Immunohistochemical staining was performed using formalin-fixed paraffin-embedded tissues from the same patient and region; no positive signal was observed by IHC in any of the investigated tissues. Spike protein detection and the time after the last vaccination are illustrated using a Likert-like scale. Cases were sorted by time post vaccination (days, for patient characteristics, see Supplementary Table 1). N/A denotes tissues not available for immunohistochemical analyses.

To examine the local spatiotemporal distribution of mRNA vaccines in humans, we first measured *BNT162b2* mRNA by qPCR and spike protein abundance by IHC in the muscle tissue at the injection site of 11 patients who died shortly after receiving *BNT162b2* vaccination. The median age of the patients was 87 years (IQR: 78–93), with 64% (*n* = 7) being female. The median time between death and cooling at 4°C was 5 days (range: 1–9; IQR: 3–6). None of the corpses showed any signs of putrefaction, neither macroscopically nor histologically. Vaccination-related and clinical baseline characteristics are provided in Supplementary Table 1. Importantly, none of the patients showed vaccine-related gross organ pathologies or death in a direct causative relationship to the vaccination (Supplementary Table 1). mRNA (#1: N/A, #02: 2.09 × 10^7^ and #04: 3.50 × 10^4^ copies/10 mg tissue) was detected by qPCR in the injection-side deltoid muscle of two of those 11 patients and in 2 of those 11 patients by in-situ hybridisation, one to three days after they received the vaccination (Fig. 2A). Spike protein expression was found in the deltoid muscles of two patients vaccinated the day before (Fig. 2A). Next, we wanted to investigate whether vaccine mRNA and spike protein could also be detected in major organs or compartments after *BNT162b2* vaccination in humans. Interestingly, *BNT162b2* mRNA levels in whole blood samples were below the detection limit under the tested conditions in all but one patient, consistent with minimal transient haematogenous dissemination observed in mice. Furthermore, neither mRNA (detected by qPCR) nor spike protein (detected by IHC) could be detected under the tested conditions in any of the major organs examined across all 11 investigated patients (Fig. 2B).

### mRNA-based vaccination leads to spike protein expression in muscle-associated fibroblasts and satellite cells, but not in antigen-presenting cells at the vaccination site in humans

**Fig. 3:**
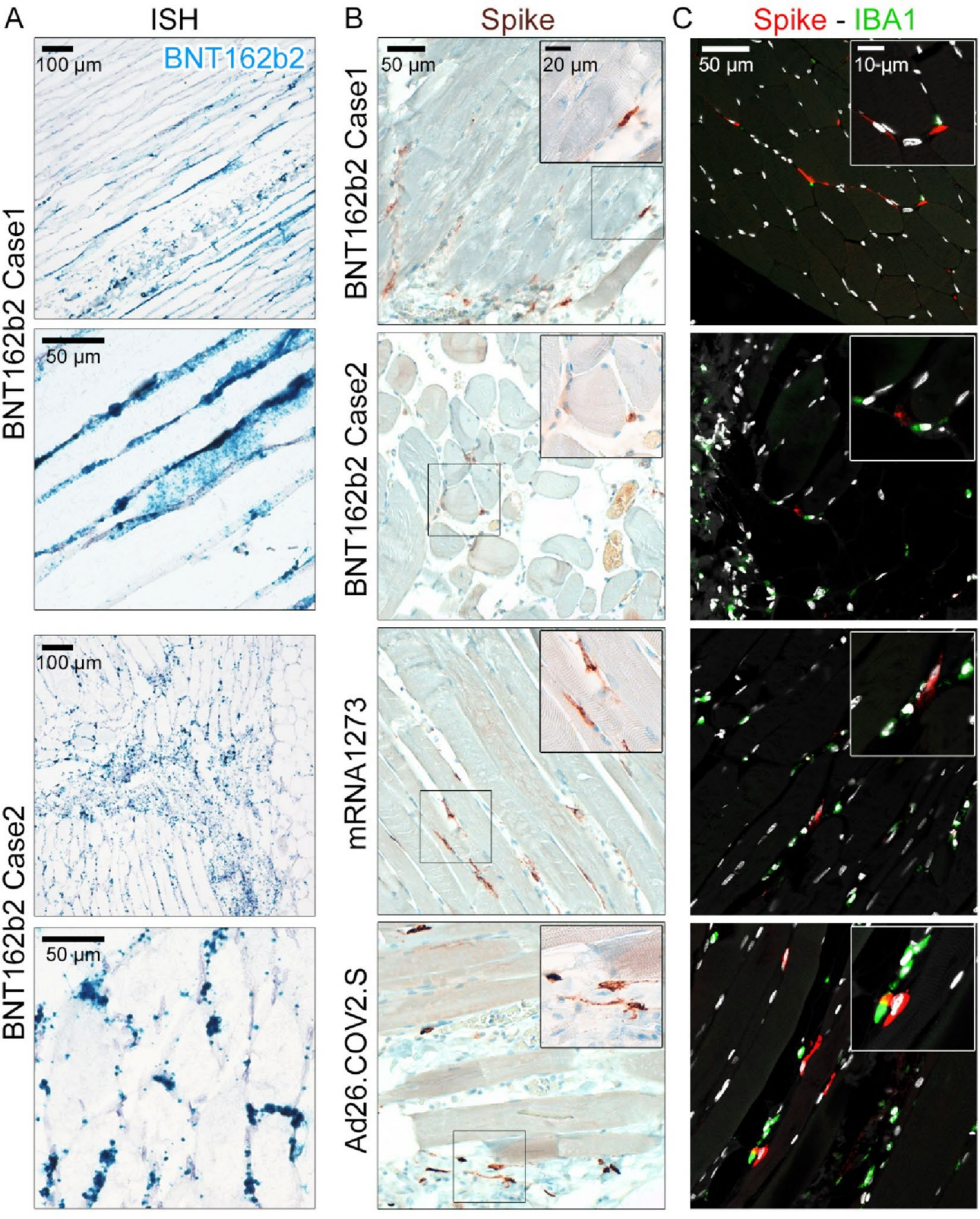
Detection of vaccine-related mRNA and spike protein in human deltoid muscle following vaccination with BNT162b2, mRNA-1273, and Ad26.COV2.S. **(A)** Distribution of BNT162b2-specific mRNA in patients 1 and 2 was determined by in-situ hybridisation (RNAscope®). (**B)** BNT162b2-, mRNA-1273-, and Ad26.COV2.S-related spike protein in the interstitial space of human deltoid muscle tissue after vaccination was detected by IHC. Note that the abundance of spike^+^-cells was higher after mRNA-1273 and Ad26.COV2.S vaccination. Boxed areas show a close-up of spike protein-positive cells. (**C)** Indirect IF analysis and confocal microscopy of co-staining for spike protein and IBA1 (tissue macrophages). Of note, spike protein^+^ cells only co-localised with IBA1^+^ cells in one patient receiving Ad26.COV2.S, but not after mRNA-based vaccination with BNT162b2 or mRNA-1273. Boxed areas show a close-up of spike protein-positive cells and their location relative to IBA1^+^ cells.

**Fig. 4:**
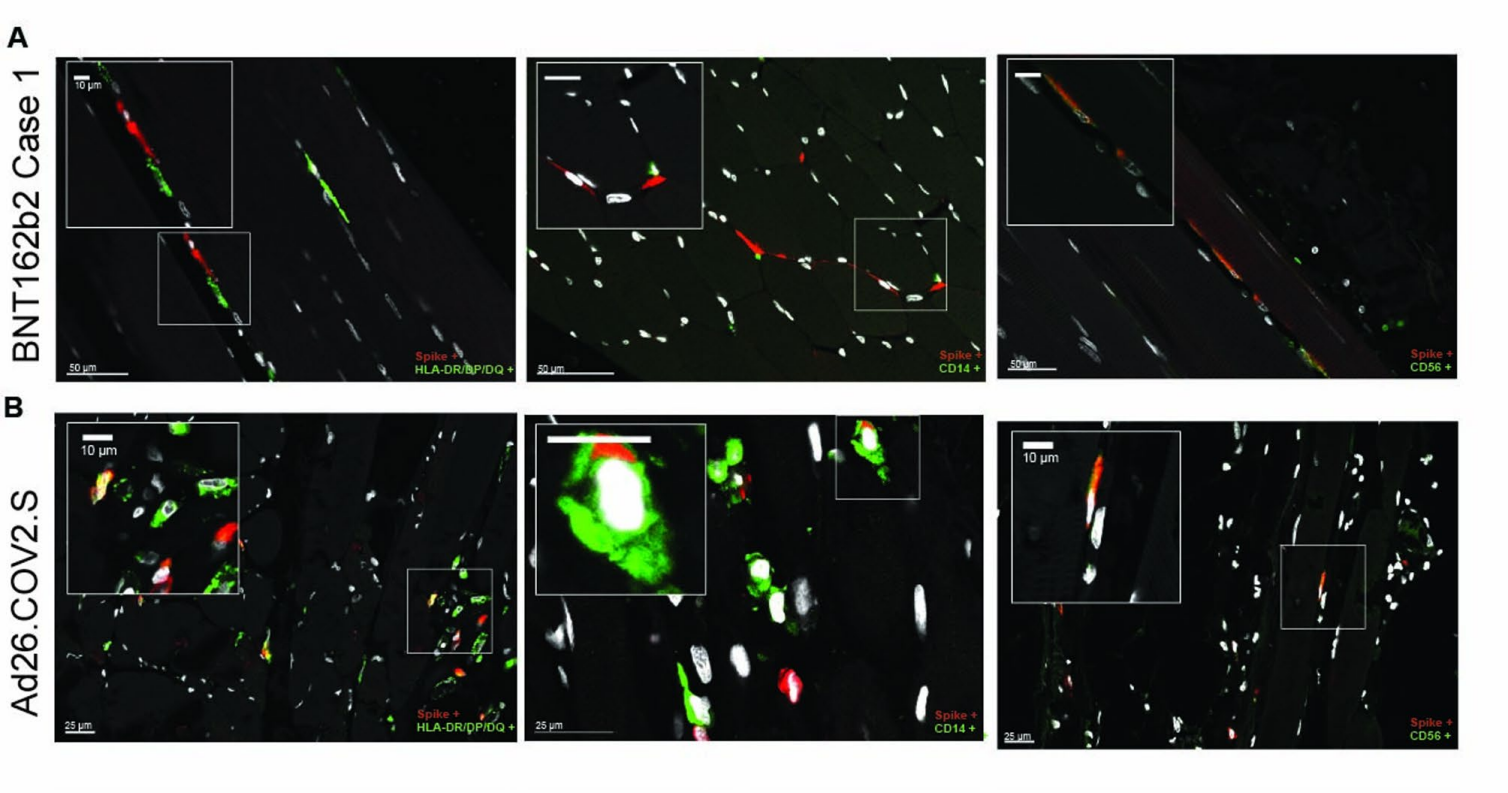
Expression of spike protein in human deltoid muscle after vaccination can be mainly detected in fibroblasts and muscle satellite cells. **(A)** BNT162b2-related spike protein in the interstitial space of the deltoid muscle tissue was detected by indirect IF analysis and confocal microscopy. Co-staining was performed to detect co-localisation of spike protein with HLA-DR/DP/DQ^+^ macrophages (left panel; green), CD14^+^ macrophages (mid panel; green), or CD56+ satellite cells (right panel; green) in patient 1. Of note, spike protein only co-localised with CD56^+^ satellite cells in this patient. Boxed areas show a close-up of spike protein-positive cells. (**B)** Muscle tissue after vaccination with Ad26.COV2.S was stained for vaccine-related spike protein in the deltoid muscle of an 89-year-old female patient, examined in a death which occured 5 days after vaccination. Indirect IF analysis and confocal microscopy show representative co-staining for spike protein and and HLA-DR/DP/DQ^+^ (left panel; green), CD14^+^ (mid panel; green), or CD56+ satellite cells (right panel; green) of this patient. Spike protein is detected in CD56^+^ satellite cells and in a subset of HLA-DR/DP/DQ^+^ cells. Of note, the engulfment of spike protein-positive cells by CD14^+^ monocytic cells was detected as well. Boxed areas show respective magnifications. Spike (red); DAPI/nuclei (white).

To examine the distribution of *BNT162b2* mRNA in muscle tissues of vaccinated patients, we used in-situ hybridisation (ISH). ISH revealed the widespread distribution of *BNT162b2*-related mRNA in the deltoid muscle of two cases; both individuals had died one day post-vaccination (Fig. 3A). *BNT162b2* RNA abundance was detected in a small number of cells in the interstitial space of the skeletal muscle (Fig. 3A). To further assess the cellular targeting of *BNT162b2* in human muscle tissue, we performed IHC to detect spike protein. Contrary to previous animal data and recent assumptions^29,28^, spike protein expression in the muscle tissue of the patients studied here did not colocalise with antigen-presenting cells in immunofluorescence and confocal microscopy, but rather with fibroblasts and CD56^+^ satellite cells (Figs. 3B and 4A). We then double-stained for macrophages (IBA1) and spike protein-expressing cells but could not detect colocalisation of both populations (Fig. 3C). Interestingly, a similar vaccine-related expression pattern of spike protein, primarily in fibroblasts and satellite cells, was observed in the deltoid muscle of an mRNA-1273-vaccinated patient two days post-vaccination (Supplementary Table 1; Fig. 3B + C). To explore the susceptibility of human muscle satellite cells in more detail, we incubated the commercially available *BNT162b2* vaccine with primary human muscle satellite cells in vitro (Figure S3B). Of note, these cells were highly susceptible to uptake of vaccine mRNA and expressed abundant amounts of vaccine-related spike protein (Figure S3B). In the patient vaccinated with mRNA-1273, the injection site beneath the skin was also sampled. Cross-sectioning and staining with an anti-spike antibody revealed that spike protein^+^-cells could also be detected at the injection site. As in the muscle tissue, the majority of spike protein^+^-cells could be identified as fibroblasts according to shape within connective and adipose tissue (Figure S5).

Taken together, *BNT162b2* and *mRNA-1273*-related mRNA and spike protein can be detected at the vaccination site of selected patients within a few days after vaccination, with satellite cells and fibroblasts being a potential human source of spike protein expression limited to a local distribution in the vaccinated muscle in our patient cohort.

### mRNA- and vector-based vaccination is associated with a local inflammatory response at the vaccination site in humans

We could validate our findings obtained from patients vaccinated with mRNA-based vaccines, when we investigated the deltoid muscle of a patient vaccinated with Ad26.COV2.S (Johnson & Johnson), an adenovirus-based vaccine encoding SARS-CoV-2 spike protein. We also included a second anti-spike antibody for detection to further validate our study (Figure S5). Indeed, a strong and even more widespread expression of spike protein compared to mRNA vaccination could be detected after vaccination with Ad26.COV2.S five days post vaccination (Fig. 3B; Figure S5). Moreover, in accordance with our findings from BNT162b2-vaccinated patients, the majority of spike protein-positive cells could be identified as spike protein^+^ fibroblasts (Fig. 3B; Figure S5) but also CD56^+^ satellite cells (Fig. 4B). Interestingly, we also found the engulfment of Ad26.COV2.S spike protein-positive cells by IBA1^+^ or CD14^+^ cells (Fig. 3C + 4B). When investigating vaccine cell targeting and expression of spike protein with immunofluorescence, spike protein colocalised with a subset of HLA-DR/DP/DQ^+^ cells in the muscle after vaccination with Ad26.COV2.S (Fig. 4B).

**Fig. 5:**
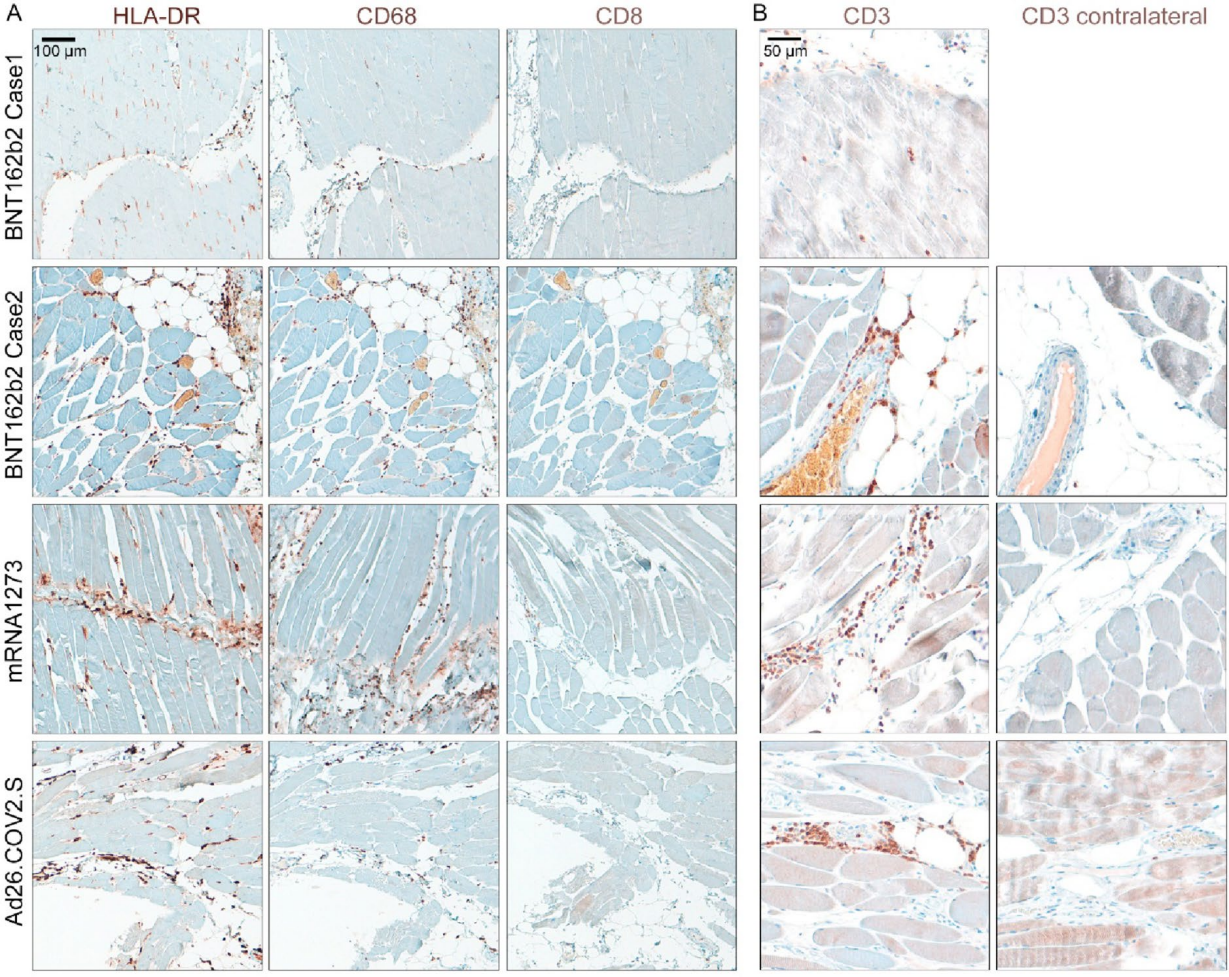
Immune infiltration in the human deltoid muscle following vaccination with BNT162b2, mRNA1273, and Ad26.COV2.S. **(A)** Human formalin-fixed paraffin-embedded muscle tissues of indicated patients were stained for HLA-DR/DP/DQ^+^ macrophages, CD68^+^ activated macrophages, or CD8^+^ T cells in BNT162b2-, mRNA1273-, and Ad26.COV2.S-vaccinated patients. While the abundance of HLA-DR/DP/DQ and CD68 was increased after vaccination, infiltration of CD8^+^ cells could not be detected at the time points investigated in both mRNA-based and adenovirus-based vaccines. **(B)** CD3^+^ T cells are highly abundant at the injection site in the deltoid muscle of indicated patients after vaccination with RNA- and vector-based vaccines. In contrast, in the contralateral muscle of the same patients, almost no T cells could be detected by IHC. The contralateral muscle tissue was not collected from patient BNT162b2 Case 1.

In line with local spike protein expression at the vaccination site, we also detected a local inflammatory response consisting of HLA-DR/DP/DQ^+^ antigen-presenting cells, CD68^+^ macrophages, but not CD8^+^ cytotoxic cells, infiltrating the muscle upon mRNA or Ad26.COV2.S vaccination (Fig. 5A). However, local inflammation with an abundance of CD3^+^ T cells at the vaccination site was observed (Fig. 5B). This immune infiltration was missing in the contralateral un-vaccinated control muscle (Fig. 5B, Figure S2D). Taken together, vaccination with mRNA-based *BNT162b2*, *mRNA-1273*, and vector-based Ad26.COV2.S leads to a local spike protein expression in human muscle tissue mainly by fibroblasts, satellite cells, and partly antigen-presenting cells and causes a sterile inflammatory response in the muscle.

## Discussion

The efficacy of mRNA vaccines has been well established through randomised controlled trials, making them a pivotal tool in combating infectious diseases such as COVID-19. This study provides insights into the distribution of mRNA vaccine-derived spike protein in mice and humans, revealing that while mRNA is initially widely distributed in the vaccinated muscle tissue, spike protein expression is primarily localised to fibroblasts and to a lesser degree to satellite cells. The accompanying infiltration of immune cells underscores a potential role for localised immune responses in vaccine-induced immunity. Our study provides comprehensive first-time data on the distribution of vaccine mRNA and vaccine-derived spike protein at the injection site in a unique cohort of deceased human individuals in temporal but not causal relation with mRNA-based COVID-19 vaccination, including one additional unique patient after immunization with a viral vector-based vaccine.

### Biodistribution of mRNA-based vaccines

To elicit an effective immune response by preventing the early degradation of mRNA and facilitating its uptake, mRNA is encapsulated in specific lipid nanoparticles, termed mRNA-lipid nanoparticles (mRNA-LNP)^30^. Early kinetic studies demonstrated that diverse application routes can lead to a local and systemic protein expression of the mRNA-encoded protein in mice^31^. In line with these previous findings, a report using radiolabelled mRNA-LNP detected lymphogenic spread of such mRNA upon intramuscular injection, not only in the muscle itself, but also to the paraaortic lymph node compartments during the first days of vaccination^16^. In a recent study, mRNA could be detected in muscle, draining lymph nodes, spleen, and liver of vaccinated rats and non-human primates upon mRNA-LNP vaccination^32^. These findings are in line with our results, which also demonstrate mRNA detection in muscle, liver and spleen in experimental mice after vaccination. More importantly, this and our study both demonstrate that mRNA detectability in the primary injection site recedes 7 days after vaccination^32^. Our study furthermore expands the organ mapping after mRNA-LNP vaccination of mice and demonstrates that after 7 days, mRNA can only be detected in the spleen of mice being vaccinated with *mRNA-1273*, but not in any other organ. Since both vaccines *BNT162b2* and *mRNA-1273* were administered to the mouse muscle in identical volumes, these differences in spatial and temporal organ distribution might be attributed to subtle differences in mRNA vaccine manufacturing such as LNP composition, nucleotide usage, cellular uptake preferences, or stability.

In humans, biodistribution studies of vaccine-derived mRNA remain scarce. A longitudinal study demonstrated that mRNA could not be detected any longer in the blood of healthy volunteers with a mean age of 42 years (range 24–70) for 28 days after mRNA-based vaccination^33^. Furthermore, another study could detect spike protein and its encoding mRNA in the draining axillary lymph nodes of vaccinated individuals for up to 60 days^34^. In our study, however, detection of mRNA in humans after vaccination was limited to the local injection site, while we could not detect mRNA in draining lymph nodes or distant organs. The median age of individuals in this study was 87 years, and many were likely affected by immunosenescence and multimorbidity. Thus, impaired immune responses may impact the long-term presence of mRNA and spike protein in this patient cohort. Interestingly, the majority of vaccine-derived spike protein, both after mRNA- and vector-based vaccination, could be detected in fibroblasts in vaccinated humans and in mice. This is in line with a recent report using single cell analysis of the injection site, which also mainly detected vaccine-related mRNA in fibroblasts^19^. Besides fibroblasts, we could identify satellite cells in the muscle tissue as a target of vaccine-derived spike protein expression. The susceptibility of satellite cells for *BNT162b2* uptake and spike protein expression was also confirmed in human satellite cells in vitro. Although we did not perform quantitative experiments here, these cells still expressed considerably more spike protein than HEK-cells when cultivated with the same amount of mRNA vaccine for the same time. Taken together, our study increases the understanding of the biodistribution of vaccine-derived mRNA by showing that in mice, early mRNA expression is predominantly present at the injection site, the blood and the lymphatic organs such as the spleen, and decreases significantly 7 days after injection. In recently deceased and mRNA-vaccinated humans, mRNA is not detectable outside of the local injection site. Importantly, the limitations of our study also need to be addressed to avoid overinterpretation of our published data. Firstly, the mice were vaccinated with a higher dosage of the mRNA vaccine per kilogram of body weight than humans normally receive. This could potentially affect the time period over which the vaccine mRNA can be detected, and increase the number of organs in which it can be detected. Secondly, the mRNA-LNP was optimised for humans, meaning that not all of the findings derived from our mouse data may be applicable to human pharmacodynamics. Third, while vaccine-associated spike mRNA and protein was measured in experimental animals without measurable delay in organ processing, our data from human vaccines were derived from post mortem tissue samples of patients who died in a temporal association with a vaccination. Unfortunately, the stability of mRNA vaccines might be affected by the latter in an organ-dependent manner, thus, our measurements might rather underestimate abundance of vaccine related spike mRNA or protein. Lastly, although based on a unique set of human tissues, the case numbers are still small and might limit the generalisability of our data.

### Local immune response after mRNA-based vaccination

Only after the emergence of COVID-19 and the widespread use of mRNA-LNPs against SARS-CoV-2 have the immune responses upon mRNA-LNP vaccination been systematically addressed^35^. To date, and supported by a wide array of studies, the systemic mechanisms of mRNA vaccination are considered relatively well understood^25,36^. Nevertheless, the local immune response at the injection site remains incompletely characterised. Even before the COVID-19 pandemic, a first study highlighted a direct local immune response in the vaccinated muscle upon mRNA-LNP vaccination, as increased chemokines and cytokines, such as IL-6, TNF, and CXCL9, could be detected^37^. Such chemokines might play a role in recruiting cells of the innate immune system to the local injection site, as another study observed an early influx of innate inflammatory cells, such as monocytes and dendritic cells, in the injected muscle of rhesus macaques that had been vaccinated with mRNA-LNP for influenza H10 hemagglutinin^38^. This study also demonstrated an early interferon-based local and systemic response signature in these animals. Newer studies underline the finding that upon mRNA-LNP injection, an influx of innate and adaptive immune cells occurs at the injection site^19^. In line with these previous reports, we could equally detect an infiltration of antigen-presenting cells, macrophages, and T cells in the injected muscle, as determined by IHC. However, we could not detect expression of spike protein by these cells. However, limitations in detection methods pose challenges to fully understand the mechanisms of mRNA vaccines. Moreover, immunohistochemistry can only detect substantial amounts of protein and may fail to identify soluble spike proteins or small quantities of protein. Interestingly, we could detect engulfment of spike-positive cells by IBA1^+^- and CD14^+^-cells in the muscle tissue of one patient five days after vaccination with the vector-based Ad26.COV2.S vaccine.

### Target cells of mRNA-based vaccines

As outlined above and suggested by multiple studies, the local immune response at the site of injection might contribute significantly to the efficacy of mRNA-LNP vaccination^39^. One important factor of this local immune response is the target cells of mRNA-LNP that facilitate the translation of the mRNA-encoded immunogenic protein. Undoubtedly, professional antigen-presenting cells, such as dendritic cells, can function in such a manner^17^. Some studies demonstrate that further infiltrating immune cells, such as macrophages, are capable of taking up mRNA-LNP and producing the mRNA-encoded protein at the site of the injection and in the draining lymph nodes^25,39^. Nonetheless, different studies also found other potential target cells of mRNA-LNP over the years. For example, one study could also detect mRNA-positive immune cells such as monocytes, T, and B cells at the local site of vaccination^16^. After a challenge with intradermally applied mRNA-LNP in human skin explants, another study found that adipocytes were the first and most abundant cell type to express the mRNA-encoded eGFP, underlining the notion that non-immune cells can also be potential target cells for mRNA vaccines^40^. This finding is in line with our study, as we have mainly detected mRNA transcripts at the injection site in non-immune cells such as fibroblasts and satellite cells. Additionally, a recent study corroborates our results, as they could detect highly enriched mRNA after vaccination with mRNA-LNP in fibroblasts at the injection site as well^19^. Mechanistically, the authors found that local mRNA-containing fibroblasts produced increased amounts of IFN-β that specifically enhanced antigen-specific cellular immune responses.

## Conclusion

This study comprehensively examines the distribution of mRNA vaccines within the human body for the first time. Neither did the clinical trial for regulatory approval examine RNA distribution systematically, nor did any other original article that we are aware of (except for one study examining mRNA presence in specific lymph nodes). Although biases may arise from postmortem degradation of vaccine RNA, the approach used in our study is the only way to systematically examine the presence of RNA in human peripheral organs. We show that vaccine RNA can be found at the injection site, but nowhere beyond, in any of the examined peripheral organs between 1 and 14 days after vaccination. Interestingly, the primary target cell type for spike protein expression was identified as fibroblasts, with satellite cells expressing the protein to a lesser degree. Of note, spike-expressing fibroblasts could also be detected at the injection site associated adipose tissue. mRNA uptake and expression of vaccine-derived mRNA in non-immune cells at the injection site after mRNA vaccination might prime and enhance the local immune environment, and mediate increased antigen protection. However, further studies need to be carried out to investigate the precise roles of fibroblast and satellite cells at the injection site after mRNA vaccination. Taken together, the involvement of different cellular subsets might be the molecular basis for different vaccine-specific immune responses and might help guide future vaccine development and optimisation efforts.

## Supplementary Information

Below is the link to the electronic supplementary material.

Supplementary Material 1

## Data Availability

All data relevant to the submission are published as part of the main manuscript or manuscript supplementary.
