# Supplementary material for: Spatiotemporal distribution of SARS-CoV-2 vaccines and vaccine-related proteins in mice and humans
Source: Sci Rep. 2026 May 19;16:15479. doi: 10.1038/s41598-026-47568-6 (PMC13187450; doi:10.1038/s41598-026-47568-6)
Supplement: Supplementary file 1 — Supplementary Material 1 [file 41598_2026_47568_MOESM1_ESM.docx]

**Spatiotemporal distribution of SARS-CoV-2 vaccines and vaccine-related proteins in mice and humans**

Fabian Heinrich^1,2,3*,#^, Jöran Lücke^4,5,6,#^, Siwen Zhang^4,5,†^, Morsal Sabihi^4,5,†^, Christian Bernreuther^7^, Katja Giersch^8^, Lena Allweiss^9,10^, Kristin Hartmann^11^, Edda Thies^11^, Philine Lange^1^, Jakob Matschke^11^, Anja A. Kühl^12^, Ronja Mothes^13^, Helena Radbruch^13^, Ann Sophie Schröder^1^, Axel Heinemann^1^, Maura Dandri^9,10^, Martin Aepfelbacher^8^, Samuel Huber^4,5^, Anastasios Giannou^4,5,6^, Markus Glatzel^11^, Benjamin Ondruschka^1^, Marc Lütgehetmann^8,10,‡^, Susanne Krasemann^11,*,‡^

^1^ Institute of Legal Medicine, University Medical Center Hamburg-Eppendorf, Butenfeld 34, 22529 Hamburg, Germany.

^2^ Department of Medical Statistics, London School of Hygiene and Tropical Medicine,

London, UK

^3^ Centre for Data and Statistical Science for Health, London School of Hygiene and Tropical

Medicine, London, UK

^4^ Section of Molecular Immunology and Gastroenterology, I. Department of Medicine, University Medical Center Hamburg-Eppendorf, Hamburg 20246, Germany.

^5^ Hamburg Center for Translational Immunology (HCTI), University Medical Center Hamburg-Eppendorf, Hamburg 20246, Germany.

^6^ Department of General, Visceral and Thoracic Surgery, University Medical Center Hamburg-Eppendorf, Hamburg 20246, Germany.

^7^ Institute of Pathology, University Medical Center Hamburg-Eppendorf, Martinistraße 52, 20251 Hamburg, Germany

^8^ Institute of Medical Microbiology, Virology and Hygiene, University Medical Center Hamburg-Eppendorf, Martinistraße 52, 20251 Hamburg, Germany

^9^ Department of Internal Medicine, University Medical Center Hamburg-Eppendorf, Martinistraße 52, 20251 Hamburg, Germany

^10^ German Center for Infection Research (DZIF), Hamburg-Lübeck-Borstel-Riems site

^11^ Institute of Neuropathology, University Medical Center Hamburg-Eppendorf, Martinistraße 52, 20251 Hamburg, Germany

^12^ Charité – Universitätsmedizin Berlin, Berlin, Germany

^13^ Department of Neuropathology, Charité-Universitätsmedizin, Berlin, Germany

**Supplementary tables and figures**

**Supplementary Table 1. Baseline sociodemographic and vaccination-related characteristics of deaths in a temporal connection with BNT162b2 vaccination (patients 1-11).** 7/11 (64%) patients received booster vaccinations. Antibodies against S1 RBD were found in 4/10 (40%) vaccines. Patients 12 and 13 were vaccinated with mRNA-1273 (Moderna) or Ad26.COV2.S (Johnson & Johnson), respectively, in a temporal connection with their death. Abbreviation: BMI, body mass index; PV, prime vaccination; BV, booster vaccination; PMI, postmortem interval; N/A, not applicable; N/D, not detected; AH, arterial hypertension; CHD, chronic heart disease; COPD, chronic obstructive pulmonary disease, PAH, pulmonary arterial hypertension; CKD, chronic kidney disease; CHF, chronic heart failure; HCM, hypertrophic cardiac myopathy; PAD, peripheral arterial disease; DCM, dilatative cardiac myopathy; S/P, status post.

| **Pat. no.** | **Age**  **[years]** | **Sex** | **BMI [kg/m^2^]** | **Time from PV to death [days]** | **Time from BV to death [days]** | **Time from PV to BV [days]** | **Cause of death by autopsy** | **Comorbidities** | **PMI [days]** | **Location of death** | **Vaccine** |
| --- | --- | --- | --- | --- | --- | --- | --- | --- | --- | --- | --- |
| 1 | 46 | F | 34·7 | 1 | No BV | N/A | Acute myocardial infarction | AH, arteriosclerosis, CHD, COPD | 1 | Home | BNT162b2 |
| 2 | 89 | M | 32·4 | 1 | No BV | N/A | Acute exacerbated COPD | Arteriosclerosis, COPD, muscular dystrophy, PAH | 2 | Home | BNT162b2 |
| 3 | 87 | F | 20·1 | 38 | 2 | 36 | Urosepsis | AH, CKD, DCM, hypothyroidism, rheumatoid disease | 9 | Home | BNT162b2 |
| 4 | 78 | M | 24·5 | 24 | 3 | 21 | Acute decompensated heart failure | Arteriosclerosis, CHF, COPD, CKD, Parkinson's disease | 6 | Nursing home | BNT162b2 |
| 5 | 93 | F | 24·4 | 25 | 4 | 21 | Acute kidney injury | AH, arteriosclerosis, CKD, COPD, dementia, type II diabetes mellitus, s/p stroke | 4 | Hospital | BNT162b2 |
| 6 | 91 | F | 29·1 | N/D | 5 | N/D | Acute decompensated heart failure | AH, arteriosclerosis, CHF, COPD, thoracic aortic aneurysm, PAD | 5 | Home | BNT162b2 |
| 7 | 68 | M | 21·5 | 26 | 5 | 21 | Aspiration pneumonia | CHD, COPD, dementia | 6 | Nursing home | BNT162b2 |
| 8 | 78 | M | 25·2 | 7 | No BV | N/A | Pericarditis | arteriosclerosis, CHF, CHD, COPD, CKD | 5 | Other | BNT162b2 |
| 9 | 94 | F | 23·8 | 29 | 8 | 21 | Aspiration pneumonia | CHF, CKD, HCM, mechanic ileus | 4 | Home | BNT162b2 |
| 10 | 82 | F | 23·1 | 13 | No BV | N/A | Acute lung edema | AH, arteriosclerosis, CKD, COPD, type II diabetes mellitus | 9 | Home | BNT162b2 |
| 11 | 95 | F | 28·0 | 35 | 14 | 21 | Acute myocardial infarction | AH, basal cell carcinoma, CHD, COPD, CKD | 3 | Nursing home | BNT162b2 |
| 12 | 64 | F | 23.4 | 2 | N/A | N/A | Subarachnoid haemorrhage | N/A | N/A | N/A | mRNA-1273 |
| 13 | 89 | F | 26.9 | 5 | N/A | N/A | Gastrointestinal infection | N/A | N/A | N/A | Ad26.COV2.S |

**
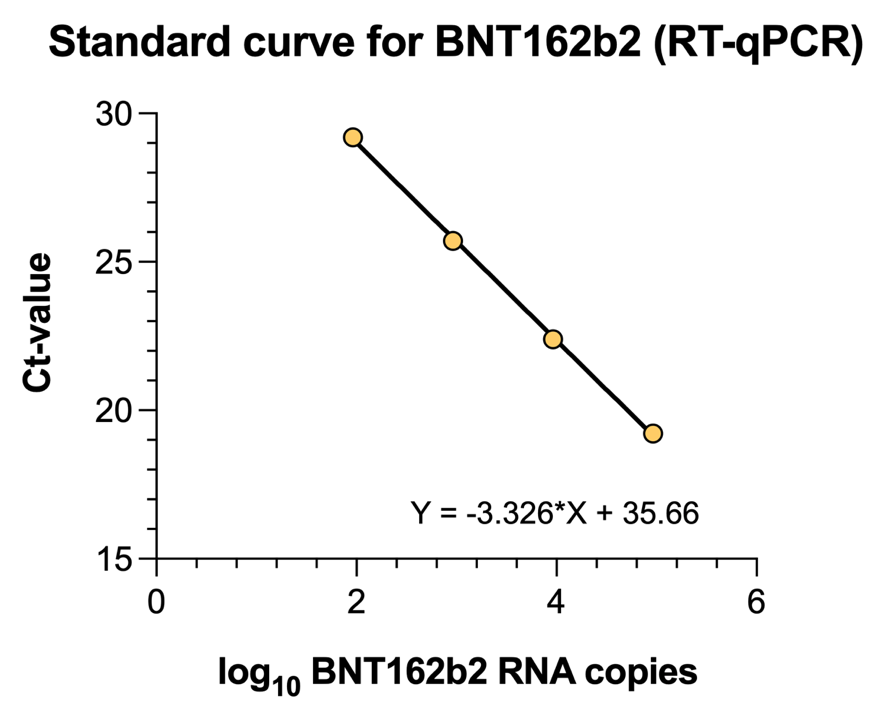
Supplementary Figure 1**

**Supplementary Figure 1. BNT162b2 RNA standard curve using the commercially** available BNT162b2 vaccine**.** The mRNA was extracted by automated nucleic acid extraction from the commercially available BNT162b2 vaccine (BioNTech SE, Mainz, Germany). BNT162b2-RNA-specific primers and probes were used (GenBank number: MT380725.1). One-step reverse transcription PCR was run on the LightCycler 480 system. A standard curve was prepared in 1:10 dilution steps (n=5). The curve formula is given.

**Supplementary Figure 2**

**
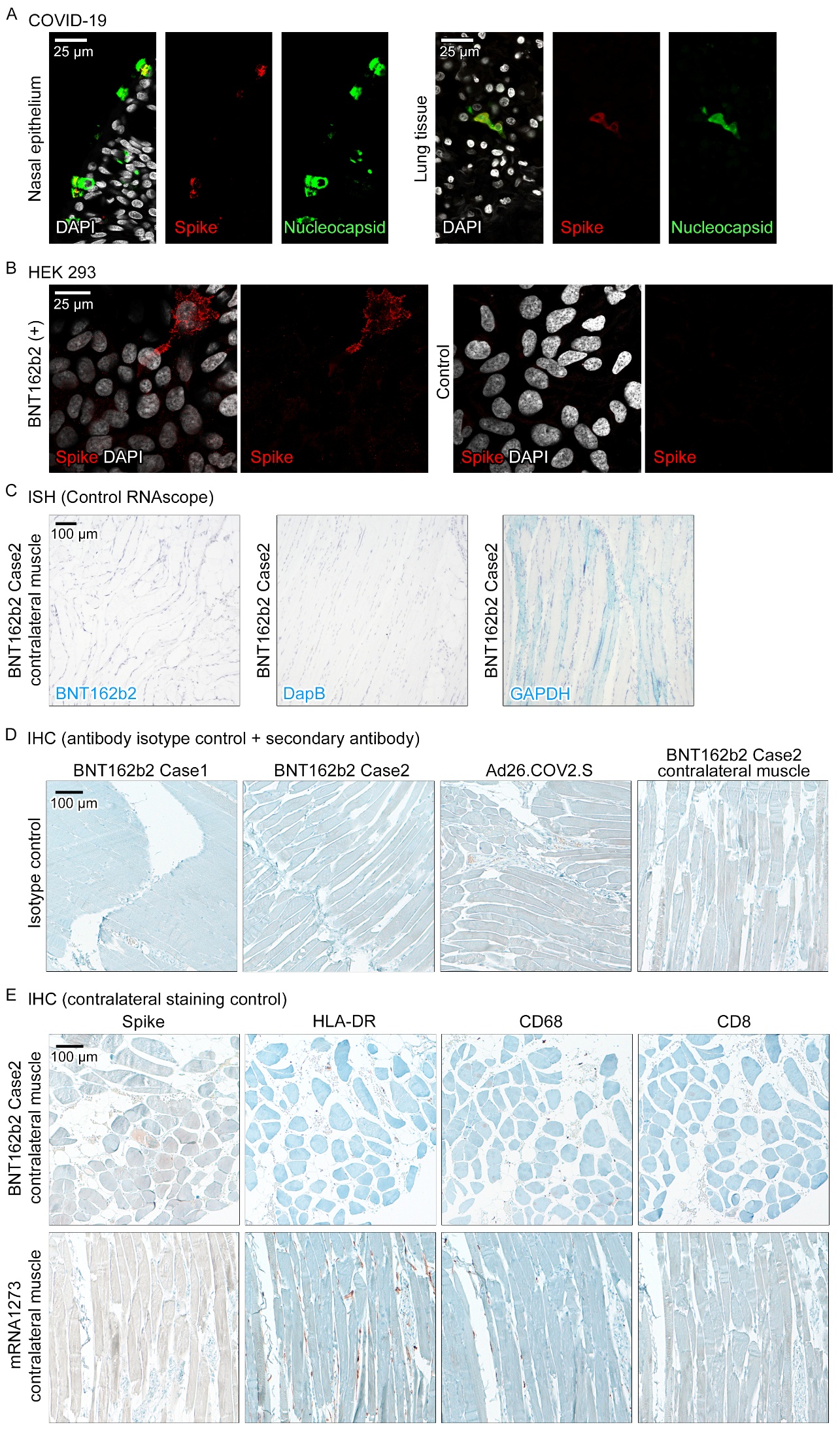
**

**Supplementary Figure 2.** **Positive and negative controls for the establishment of anti-spike antibodies and the detection of spike protein by *in-situ* hybridization and immunohistochemistry.** **A.** To confirm the specificity of the anti-SARS-CoV-2 spike protein directed antibody (red), we performed double staining with an anti-SARS-CoV-2 nucleocapsid protein directed antibody (green) in nasal epithelium and lung tissue of a patient with fatal COVID-19. Staining overlay showed positive co-localization of SARS-CoV-2 positive cells with both antibodies with proper sub-cellular localization of spike protein; nuclei (DAPI) are depicted white. **B.** To evaluate the detection of vaccine-related spike protein using anti-spike antibodies, human HEK cells were incubated with the commercially available BNT162b2 vaccine for 48h. Subsequently, indirect immunofluorescence and confocal microscopy were performed as described. Specific spike signal (red; anti-SARS-CoV-2 Spike S2 #GTX632604) can be detected in cells incubated with (left) but not without (right) BNT162b2; nuclei (DAPI) are depicted white. The antibodies with the best signal-to-noise ratio were utilized for all further experiments in tissues. **C.** Positive and negative controls for *in-situ* hybridization (ISH) were taken from patient 2. **D.** Formalin-fixed paraffin-embedded human muscle tissues from injection sites of several patients were stained with an isotype control antibody. No staining could be detected, underscoring the specificity of the anti-spike protein antibody used in our study. **E.** Contralateral muscle sections of two deceased individuals were stained with anti-spike antibody to confirm specificity, and moreover with diverse anti-immune cell antibodies using immunohistochemistry. In contrast to the vaccine-injected deltoid muscle, neither spike protein nor activation or recruitment of immune cells could be detected.

**Supplementary Figure 3**


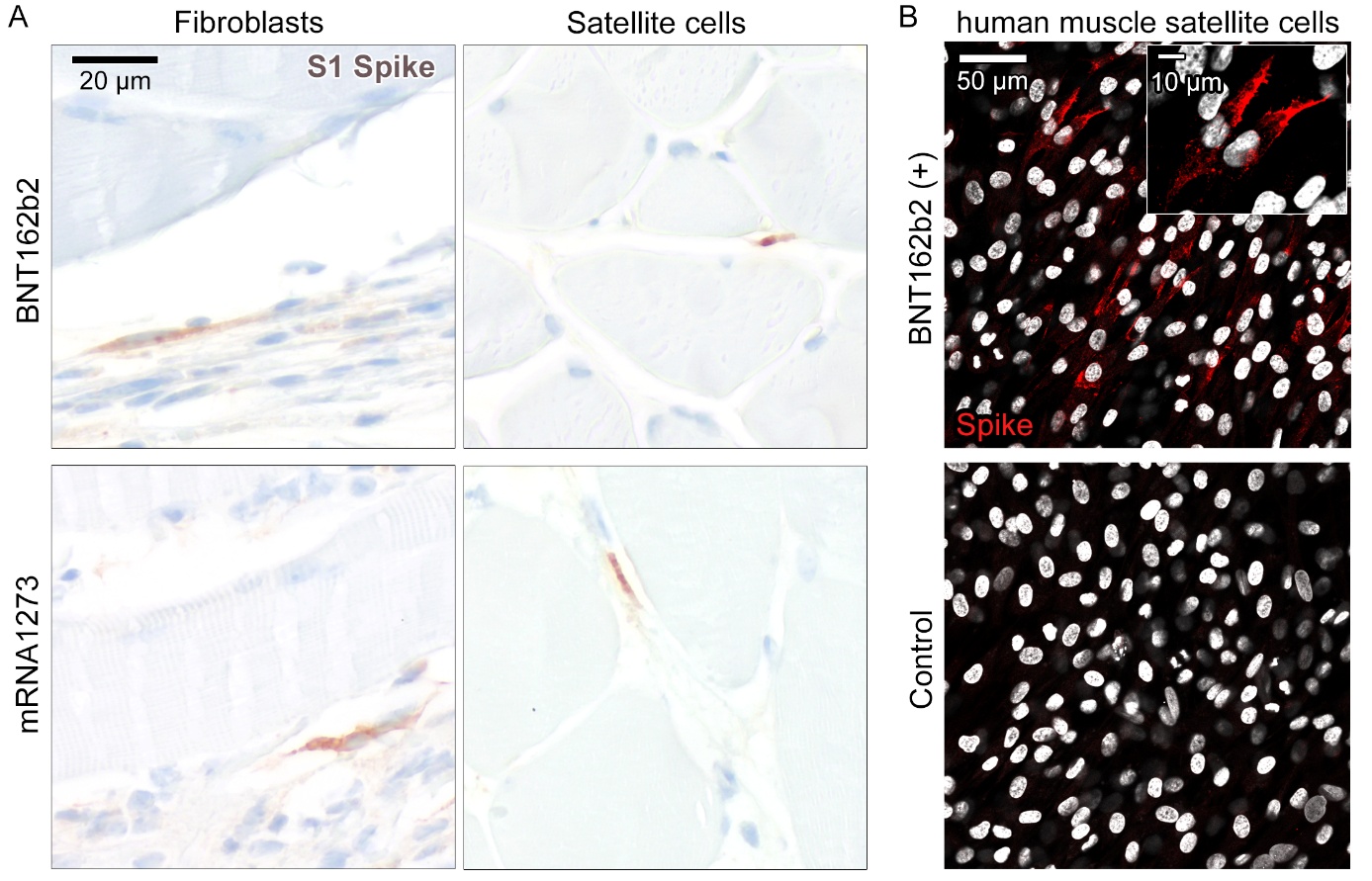


**Supplementary Figure 3. Expression of the vaccine-associated spike protein can be mainly detected in fibroblasts and satellite cells in a mouse model and *in vitro*.** **A.** Representative close-ups of immunohistochemical detection for S1 spike protein in muscle tissue of mice one day after vaccination with BNT162b2 or mRNA-1273 displayed that spike protein could be mainly found in muscle-associated fibroblasts and satellite cells. **B.** Incubation of primary human muscle satellite cells *in vitro* for 48h with commercially available BNT162b2 vaccine showed the high susceptibility of these cells towards uptake of vaccine mRNA and expression of vaccine-related Spike protein (Spike in red; DAPI/nuclei in white).

**Supplementary Figure 4**


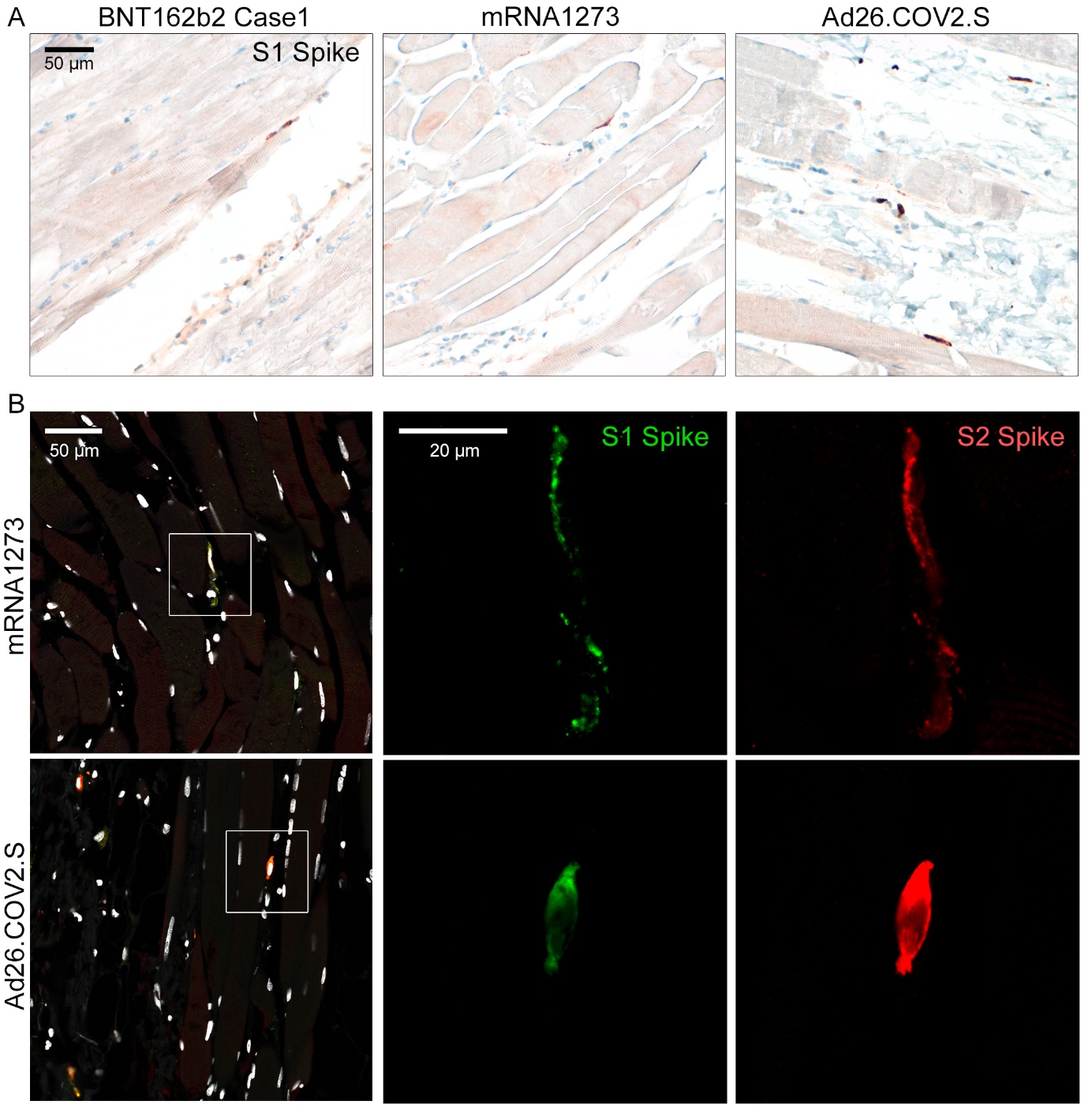


**Supplementary Figure 4. S1 and S2 spike protein can be detected in human deltoid muscle after vaccination with mRNA- and vector-based vaccines by immunohistochemistry.** **A.** To confirm the expression of vaccine-related spike protein in the human deltoid muscle, we used an alternative antibody (#GTX135356) detecting the S1 subunit of the spike protein. Representative images of spike S1 after vaccination with BNT162b2, mRNA1273, or Ad26.COV2.S show comparable abundance and staining pattern to S2 expression (see Figure 3B). **B.** Fluorescence double staining of S1 and S2 spike protein in human deltoid muscle after vaccination with mRNA1273 and Ad26.COV2.S showed a slightly different sub-cellular distribution of both spike protein subunits as expected for proper processing of the vaccine-related spike protein.

**Supplementary Figure 5**


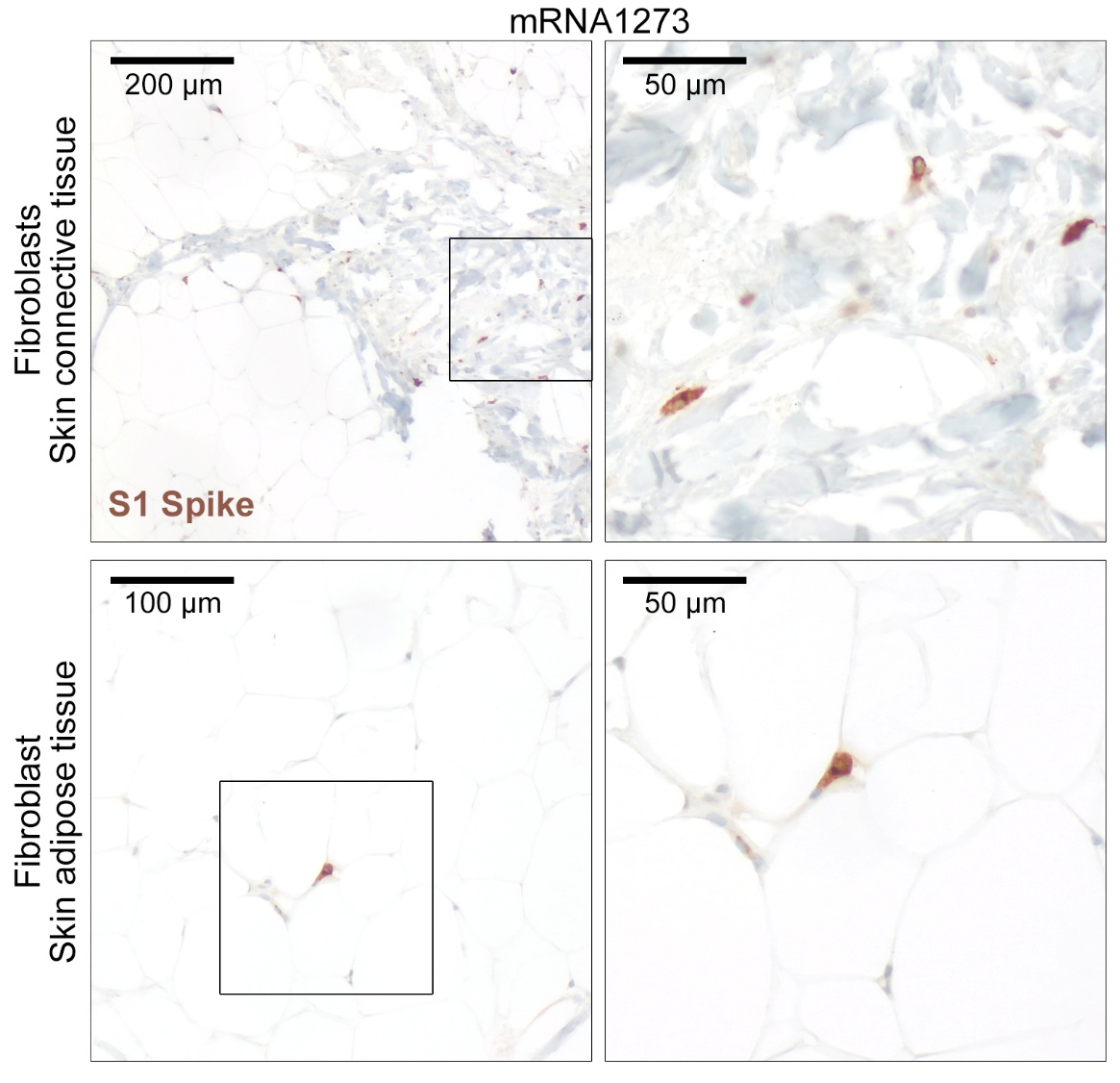


**Supplementary Figure 5. Extra-muscular expression of vaccine spike protein in adipose and connective tissue at the injection site.** **A.** For one patient, the injection site (skin and underlying tissue) after vaccination with mRNA-1273 could be sampled and was investigated by IHC for the abundance of vaccine-related spike protein. Interestingly, as in human deltoid muscle tissue, spike protein could be mainly detected in fibroblasts in skin-associated connective tissue and adipose tissue after vaccination with mRNA-1273.
